# Auditory gamma-band entrainment enhances default mode network connectivity in dementia patients

**DOI:** 10.1038/s41598-024-63727-z

**Published:** 2024-06-07

**Authors:** Mojtaba Lahijanian, Hamid Aghajan, Zahra Vahabi

**Affiliations:** 1 Department of Electrical Engineering, Sharif University of Technology, Tehran, Iran; 2 Department of Geriatric Medicine, Ziaeian Hospital, Tehran University of Medical Sciences, Tehran, Iran

**Keywords:** Alzheimer's disease, Network models

## Abstract

Dementia, and in particular Alzheimer’s disease (AD), can be characterized by disrupted functional connectivity in the brain caused by beta-amyloid deposition in neural links. Non-pharmaceutical treatments for dementia have recently explored interventions involving the stimulation of neuronal populations in the gamma band. These interventions aim to restore brain network functionality by synchronizing rhythmic energy through various stimulation modalities. Entrainment, a newly proposed non-invasive sensory stimulation method, has shown promise in improving cognitive functions in dementia patients. This study investigates the effectiveness of entrainment in terms of promoting neural synchrony and spatial connectivity across the cortex. EEG signals were recorded during a 40 Hz auditory entrainment session conducted with a group of elderly participants with dementia. Phase locking value (PLV) between different intraregional and interregional sites was examined as an attribute of network synchronization, and connectivity of local and distant links were compared during the stimulation and rest trials. Our findings demonstrate enhanced neural synchrony between the frontal and parietal regions, which are key components of the brain’s default mode network (DMN). The DMN operation is known to be impacted by dementia’s progression, leading to reduced functional connectivity across the parieto-frontal pathways. Notably, entrainment alone significantly improves synchrony between these DMN components, suggesting its potential for restoring functional connectivity.

## Introduction

As a neurodegenerative disease, dementia due to Alzheimer’s disease (AD) involves the brain via different mechanisms. The accumulation of insoluble plaque called beta-amyloid (Aβ) outside neurons, and neurofibrillary tangles called phosphorylated tau inside neurons, are known as AD’s major characteristics which lead the brain to a functionally deteriorative state and cause loss of synapses^1–3^. As these neuropathological effects advance, signs of network operation deficiencies appear. Brain rhythms get out of order, and neuronal synchrony of brain oscillations becomes disheveled, causing structural and functional changes in the brain’s neuronal networks^4^. As a major manifest of network disruption, the efficacy of inhibitory gamma-band activity diminishes^5–7^, excitatory neurons act superfluously^1,8,9^, leading to further acceleration of Aβ production as a byproduct of neural activity^10^ and further advancing the cycle of the disease progress. Much of the oscillatory abnormalities associated with AD commence in areas such as the entorhinal cortex, the limbic system, and the hippocampus, which are responsible for cognition, memory tasks, and sensory processes, and known to involve high-frequency oscillatory activity in the gamma band^6,8,11^.

Neurodegeneration, network alteration, and cognitive deficit are all manifested in AD spectrum^4,5,12^, which informs the potential correlation that exists between them. Although definite cause and effect links governing the relationships among these phenomena need to be yet clarified, much of the initial approaches to AD treatment have targeted neurodegeneration or symptom relief through pharmaceutical remedies. Most notably, the newly FDA-approved drugs Aducanumab^13^ and Lecanemab^14^ are reported to reduce the Aβ load in the brain. However, the much debated clinical trials of Aducanumab have fallen short in proving noticeable benefit in improving cognitive symptoms of dementia^1,15^, and even its efficacy in lowering Aβ is questioned^16^, extending the controversy about it to a span from potential safety risks and high cost to the accelerated approval process that relied on this surrogate endpoint and not cognitive improvements^17,18^. As for the newer drug Lecanemab, while moderate slowing of cognitive decline has been observed in its clinical trial^14^, its safety is still under serious question as adverse side effects such as cerebral hemorrhage has been reported after its administration^19^. Besides the high cost of both drugs and the challenges associated with treatment regimens involving frequent hospital visits to receive infusions of the drug, it has also been reported earlier that while reducing the Aβ load can slow the progression of AD, even a great reduction in Aβ levels may not lead to memory retrieval improvement^1^. The shortcomings of the current remedial approaches to AD treatment may stem from ignoring the intricate interactions between the long distance and local synaptic damage inflicted by Aβ aggregation and the large-scale network alterations that shift the operating point of feedforward and feedback neuronal networks.

It is still a matter of debate whether network alterations such as deficits in gamma activity are a consequence of the underlying neurodegenerative processes or they indeed play a causal role in inducing neuropathological changes that promote the disease progression. While neuropathological effects such as the aggregation of surplus Aβ in intercellular space and the ensuing synaptic loss are reported to cause hyperexcitability in excitatory neural populations leading to network disruption^1,8^, other evidences point to the presence of network alterations such as reduced gamma oscillations, decreased cross-frequency coupling activity, and diminished functional connectivity in the default mode network (DMN) even before plaque aggregates are ostensibly detectable^20–26^. The DMN is one of the most vulnerable networks affected by Aβ deposits in the early stages of the disease^27–29^. This network involves the circuitry of the frontal and parietal cortices and is known to be activated in various memory functions^24,27,28^. The normal operation of the DMN is affected by normal ageing due to weakened long distance connections, and dementia exacerbates such connectivity loss^23–25,30^. Based on these observations as well as the more recent evidence suggesting a causal direction from the quality of brain oscillations to the performance of memory and attention functions^31,32^, approaches to mitigate or compensate for network alterations manifested in the lowered quality of connectivity in the brain’s oscillatory activity can serve as alternative pathways to AD treatment. Detailed examination of the changes in the brain’s oscillatory attributes such as the quality of synchronization instilled through such approaches can offer insight into the interwoven cycles of cause and effect involving neuropathological changes and network alterations, paving the way for more integrative approaches to the treatment of the disease.

Interventions based on stimulating neural populations in the gamma band have been recently proposed and are under examination on animal models of AD^20,33–35^ and in human studies^26,36–44^. These approaches aim to reinvigorate the functionality of the brain networks through synchronized pumping of rhythmic energy via one or multiple sensory modalities. Stimulating neuronal networks of the brain by an external stimulus at a specific frequency drives the neurons to undergo oscillatory spiking behavior in that frequency, which is referred to as entrainment^45^. The idea behind such network training approaches is to compensate for the functional weaknesses caused by synaptic loss due to Aβ plaque deposition. These methods primarily focus on combating the network alteration characteristics of AD, and early results have shown promising outcomes in improving cognitive functionality of the subjects. For example, optogenetically-induced 40 Hz entrainment of the Parvalbumin (PV) inhibitory interneuron cells in the hippocampus has been reported to reduce the level of amyloid plaque in different mouse models of AD^20,35^. Non-invasive entrainment of gamma oscillations using auditory and visual stimulation has been shown to improve memory performance in AD mouse models and more interestingly, reduce the amyloid load in the auditory cortex, hippocampus, and the medial prefrontal cortex of the mice^20,33^. Exposure to flickering light at 40 Hz has been shown to produce a similar effect in the visual cortex^20,34,35^. Remarkably, treating the AD mouse models with repeated entrainment sessions has been reported to result in improved memory and cognitive functions and slowing the neurodegenerative effects of AD^20,33–35^. The induction of gamma oscillations in AD mouse models through sensory stimulation has been shown to also improve neuroimmune biochemical signaling known to promote microglia activity^46^ and enhance their Aβ uptake^20^. Meanwhile, long-term application of non-invasive 40 Hz stimulation has demonstrated improved rhythmicity of daily living activity, sleep, and memory for dementia patients alongside enhanced functional connectivity in the DMN^43,47,48^. Furthermore, even a single auditory entrainment session has been found to enhance cognitive performance, working memory tasks, and emotional states in healthy participants^42,44^.

In this study, we aim to examine the effects of auditory entrainment on improving the synchronization characteristics of the brain oscillations within local areas of the frontal and parietal regions as well as between these regions, and thereby provide an explanation for network-level connectivity enhancement induced by entrainment. Synchrony in the activity of the brain’s neuronal networks signifies the consistency of temporal or spatial fluctuations within and between the networks involved in the DMN, and is considered as a surrogate measure of communication or functional connectivity with implications on the performance of cognitive tasks^49^.

Based on EEG data recorded during 40 Hz auditory stimulation of a group of elderly participants suffering from dementia, a metric is defined for the quality of the entrainment response which places the participants over a spectrum from least responsive to most responsive to the target stimulation frequency of 40 Hz. We identify the phase locking value (PLV) as an essential attribute of network synchronization to measure the connectivity of different sites over the scalp and compare the quality of connectivity between the stimulus and resting-state trials. After investigating synchronization values within the frontal region (F2F), between the frontal and parietal regions (F2P), and within the parietal region (P2P), we show that 40 Hz entrainment significantly increases the amount of synchrony between all sites of the three sets of connections. Specifically, we observe that the entrained oscillatory activity of F2P sites, representing long-range parieto-frontal connections, exhibits the most significant increase in synchronization compared to the other two sets involving local connections within the frontal or parietal regions. Moreover, the quality of entrainment correlates with the level of synchronization observed within each set. By localizing the sources of oscillatory activity in the brain, we observe that the most entrained sources predominantly reside in areas overlapping with the default mode network. The observed improvement in long-range communication synchrony involving the frontal and parietal regions, which are the core sites of the DMN, along with the presence of entrained brain sources within this network, reflect the recovery of functional connectivity in the DMN and may help explain the cognitive improvements observed in response to gamma entrainment. By elucidating the effects of gamma entrainment on the synchronization of oscillations across the brain, our study contributes to understanding the therapeutic potentials of sensory stimulation of the brain as a non-pharmaceutical and non-invasive intervention approach for the treatment of dementia.

## Results

### Variability in entrainment response: assessing individual responsiveness to stimulus-induced brain synchronization

**Figure 1:**
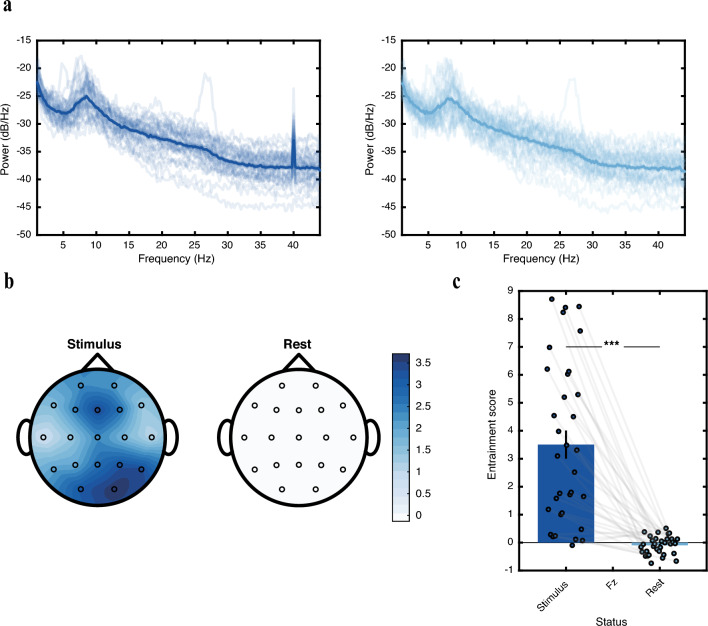
Entrainment occurrence and entrainment score distribution during stimulation and rest cycles. (**a**) Power spectral density (PSD) of the entire task averaged over all trials and channels for stimulus (left) and rest (right) cycles. The thick line represents the mean PSD calculated across all participants, while the shadow lines correspond to the PSD of each individual. The peak at 40 Hz during the stimulus cycle indicates the population’s response to the stimulus. (**b**) Topographic distribution of entrainment score (ES) over the scalp, averaged across the entire population during stimulus (left) and rest (right) cycles. The frontal, parietal, and occipital channels exhibit the highest levels of entrainment scores. ES reflects the quality of entrainment at the target frequency (40 Hz) relative to the surrounding frequencies. (**c**) ES calculated from the Fz channel during stimulus and rest cycles. The height of the bar plots represents the mean ES value between participants during each interval, while the black lines indicate the standard error of the mean. Empty circles represent individual participants, and the gray line connects paired samples (same participant) in the two intervals. ***, p < 0.001.

There is no standard definition for an entrained brain. While stimulating the brain with 40 Hz auditory or visual stimuli is referred to as entrainment, our results show that such stimulation does not necessarily cause notable entrained oscillatory activity in all brains. Based on earlier studies on entrainment^26,37,50^, a clear response of the brain to the stimulant frequency is expected as a peak in the spectrogram of the recorded response on that frequency. In Fig. 1a, we present the power spectral density (PSD) for each participant, averaged across all channels and trials, during both the stimulus and rest intervals. Notably, there is a significant increase in power at the frequency of the entraining stimulus during the stimulus interval, which indicates the population's response to the external stimulus.

Based on these observations, we propose a method to quantitatively assess the degree of brain entrainment resulting from external stimulation. Figure 1b demonstrates that entrainment is primarily observed in the frontal, parietal, and occipital channels, with F3, Fz, F4, P3, Pz, P4, O1, and O2 exhibiting the highest levels of entrained responses. Importantly, it is noteworthy that auditory stimulation alone can induce entrainment in a significant portion of the brain across the entire participant population (general demographic information of participants is included in Table 1 in Supplementary Material).

Given that previous entrainment studies have associated the activity of the frontal lobe with improved cognitive functions^33–35^, we focus on the entrained response at the Fz channel during both the stimulus and rest intervals to investigate the quality of entrainment among participants, as depicted in Fig. 1c. We introduce the entrainment score (ES) as a measure of the level of entrainment over each channel (see “Methods” for details). Our analysis reveals a significant difference in the ES for channel Fz between the stimulus and rest intervals (paired t-test, t_32_ = 6.9475, p-value = 7.2124e−08). Furthermore, the distribution of the ES during the stimulus interval demonstrates that not all participants exhibit equally strong responses to the stimulus, resulting in a range of response powers. To further analyze these variations, we assign an overall entrainment response (ER) index to each participant, indicating their degree of responsiveness based on the ES difference between rest and stimulus. This analysis allows us to assess and compare the quality of entrainment across individuals and compare them to the baseline.

### Entrainment-induced synchronization and connectivity in frontoparietal networks

**Figure 2:**
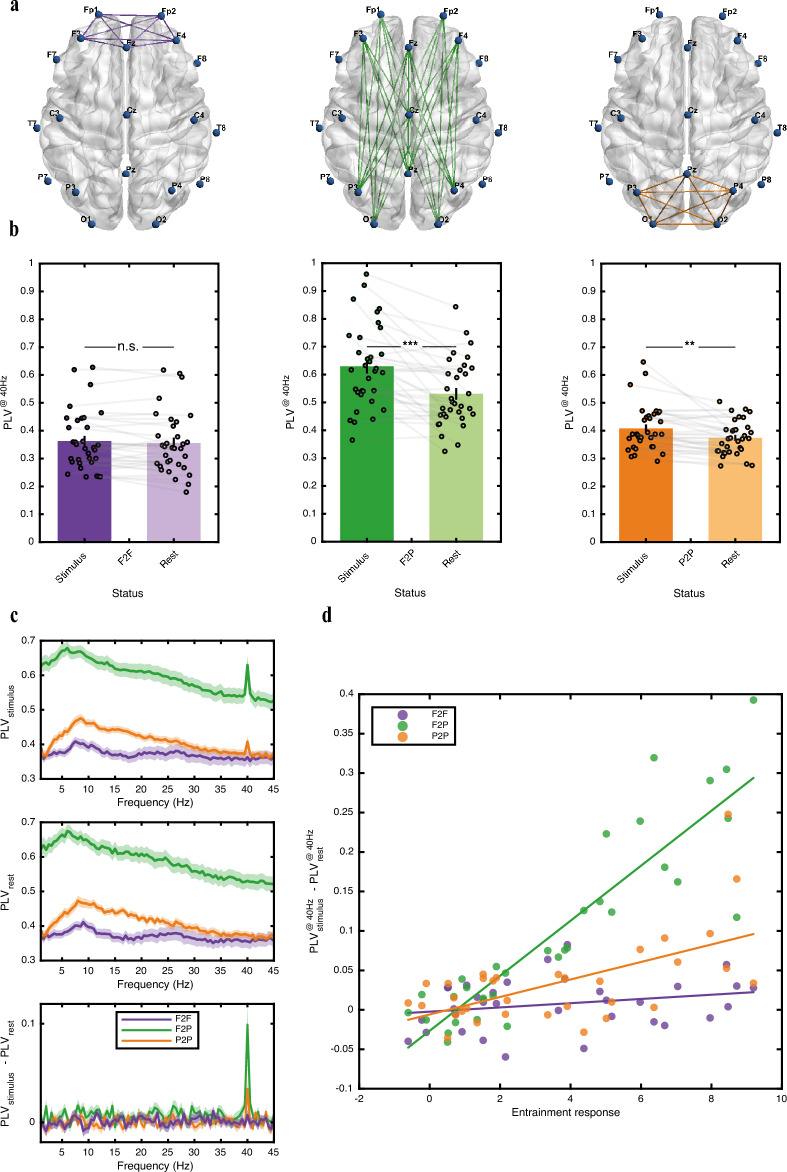
Bipolar site representation and phase locking value (PLV) analysis. (**a**) Bipolar site configuration for frontal to frontal (F2F, left), frontal to parietal (F2P, middle), and parietal to parietal (P2P, right) connections. The colored lines indicate the corresponding two unipolar channels associated with each bipolar site. (**b**) PLV calculated at the 40 Hz frequency bin for each paired site within the F2F (left), F2P (middle), and P2P (right) sets of sites. The height of the bar plots represents the average PLV of participants paired between the stimulus and rest intervals. The black lines indicate the standard error of the mean. Empty circles represent individual participants, and the gray line connects paired samples (same participant) during the stimulus and rest intervals. **, p < 0.01; ***, p < 0.001. n.s.: not significant. (**c**) The PLV calculated across a spectrum of frequency bins (0.5 Hz width), ranging from 1 to 45 Hz. The average value for bipolar sites in each set of sites is calculated during the stimulus (upper panel) and rest (middle panel) cycles, with the lower panel showing the differences between the stimulus and rest values. The solid line indicates the mean PLV for all participants, while shadow lines represent the standard error of the mean. Notably, the sharp peak at 40 Hz in the lower panel highlights the stimulus-driven connectivity enhancement. This enhancement is most prominent in the distant F2P connections, followed by the local P2P and F2F site sets. (**d**) Correlation between entrainment quality and synchronized activity within site sets. Entrainment response (ER) serves as an indicator of a participant’s entrainment during the stimulus cycles relative to the resting-state baseline activity. ER is strongly correlated with the improvement in phase synchronization from the rest to stimulus intervals, as measured by the phase locking value (PLV) difference, averaged across paired sites belonging to the frontal to parietal (F2P) set (corr. coeff. = 0.894). A moderate positive correlation is also observed between phase synchronization difference in parietal to parietal (P2P) set and ER (corr. coeff. = 0.595). Additionally, a slight positive correlation is observed between ER and the averaged phase synchronization difference in frontal to frontal (F2F) set (corr. coeff. = 0.247). Each data point represents an individual participant.

For our subsequent analysis, we focus on the frontal and parietal regions of the brain, which exhibit a higher degree of entrainment (Fig. 1b). Our objective is to examine the level of synchronization within and between these two areas as an indicator of the functional connectivity of local and distant brain regions. To assess this, we consider the frontal and parietal channels and evaluate the bipolar sites defined by taking the differences between selected unipolar channels in these regions. Specifically, the frontal-to-frontal (F2F) sites consist of unipolar channels selected from the frontal area. Similarly, the parietal-to-parietal (P2P) sites are formed using channels chosen from the parietal region. Additionally, the frontal-to-parietal (F2P) sites are configured with one unipolar channel from the frontal region and another from the parietal region. Figure 2a visually represents bipolar sites for each group, showcasing the associated unipolar channels. The corresponding summary in Table 2 in Supplementary Material details the specific unipolar channels subtracted from each other to generate bipolar sites, providing further clarity.

In the following, we investigate whether the entrained brain oscillatory activity recorded from each pair of sites within the F2F, F2P, and P2P groups maintains persistent synchronization across specific frequency bins. To assess this, we calculate the phase locking value (PLV) for all site pairs during the stimulation and rest cycles. The frequency bins, each with a width of 0.5 Hz, range from 1 to 45 Hz. PLV calculates how two oscillatory signals fluctuate congruently by temporal averaging of their phase-locking vectors. In our study, we utilize PLV as a measure to evaluate the spatial coupling of brain oscillatory activity resulting from entrainment and to examine the functional connectivity between the frontal and parietal regions.

Figure 2c illustrates the average connectivity between bipolar sites in each set (F2F, F2P, and P2P) during the stimulus (upper panel) and rest (middle panel) cycles. Notably, even during rest, there is a substantial amount of synchronization between F2P sites compared to the other sets across all frequency bins. It is worth noting that the default mode network, which plays a crucial role in intrinsic brain activity during rest, encompasses the interactions between these two brain regions. Additionally, a distinct peak is observed in the theta band for F2P connectivity. In contrast, for the P2P and F2F sites a peak in connectivity occurs in the alpha band. It is noteworthy to point out the presence of these peaks during both stimulus and rest cycles, indicating a lack of effect due to the stimulation. To further examine this assessment, the difference in PLV from rest to stimulus is plotted for all frequency bins in the lower panel of Fig. 2c. This plot underscores that only the 40 Hz enhancement in connectivity is stimulus-driven. According to this plot, F2P sites exhibit the highest enhancement in connectivity, followed by P2P and F2F.

In the subsequent analysis, our attention narrows to the stimulus-driven connectivity specifically at the 40 Hz bin, aiming for a detailed exploration of the target frequency’s role in enhancing connectivity. As previously mentioned and depicted in Fig. 2b, the F2P sites exhibit higher levels of synchronization even during the rest intervals compared to both F2F and P2P areas. When comparing the PLV at 40 Hz during the rest intervals between each set of sites, we observe a statistically significant higher level of synchronization in the long-range F2P connections compared to both the local connections in F2F and P2P sites (paired t-test, t_32_ = 8.9443, p-value = 3.2280e−10 for comparison of F2P with P2P, and t_32_ = 5.8607, p-value = 1.6253e−06 for comparison of F2P with F2F during rest; Fig. 2b).

Furthermore, we examine how entrainment influences the level of synchronization from the resting-state to stimulus-driven activity in all sets of connections, noting that the highest increment occurs for the F2P sites. Figure 2b demonstrates a significant increase in the PLV from rest to stimulus for the F2P sites (paired t-test, t_32_ = 4.8957, p-value = 2.6901e−05; Fig. 2b center). Similarly, the P2P sites exhibit a significant increase in synchronization from rest to stimulus (paired t-test, t_32_ = 3.5254, p-value = 0.0013; Fig. 2b right). However, the slight increment in PLV from rest to stimulus among the F2F sites is not statistically significant (paired t-test, t_32_ = 1.3039, p-value = 0.2016; Fig. 2b left).

### Individual-level analysis reveals differential impact of entrainment on synchronization across frontal and parietal regions

We now examine the impact of entrainment on synchronization at the individual level. While participants vary in their entrainment patterns, we rank them based on their level of entrainment using the ER index. The relationship between the ER value and the enhancement in synchronization from rest to stimulus for each participant and sets of sites is depicted in Fig. 2d. The correlation between the ER value and the difference in PLV at 40 Hz between rest and stimulus highlights that stronger entrainment leads to greater improvement in synchronization among paired sites in all sets of connections (F2F, F2P, and P2P). Notably, the effect of entrainment is most pronounced in long-distance F2P sites compared to the local P2P and F2F sites. A significant positive correlation is observed between F2P connectivity and the ER value from rest to stimulus (corr. coeff. = 0.894, p-value = 2.5737e−12), suggesting that increased gamma power due to entrainment facilitates synchronized activity between the frontal and parietal regions. Similarly, a moderate correlation is observed between P2P synchronization and the ER value (corr. coeff. = 0.595, p-value = 2.6000e−04), indicating the influence of entrainment on improving intraregional connections within the parietal area. However, in the case of F2F sites, there is no significant correlation between entrainment-induced synchronization and the ER value (corr. coeff. = 0.247, p-value = 0.1667), suggesting a different pattern of synchronization dynamics for this site. Nevertheless, the positive correlation suggests a subtle effect of entrainment in improving synchronization among paired sites within this region. Overall, our findings demonstrate the differential impact of entrainment on synchronization across local and distant brain regions.

### Effect of entrainment on default mode network connectivity: insight from source localization

Given the observed significant improvements in frontoparietal (F2P) connectivity and the involvement of the frontal and parietal regions in the default mode network (DMN), we now set to investigate whether entrainment primarily and effectively influences the DMN network. To gain further insight about the location of the entrained oscillatory activity within the brain relative to the areas involved in the DMN, we apply blind source separation techniques to the recorded EEG data.

**Figure 3:**
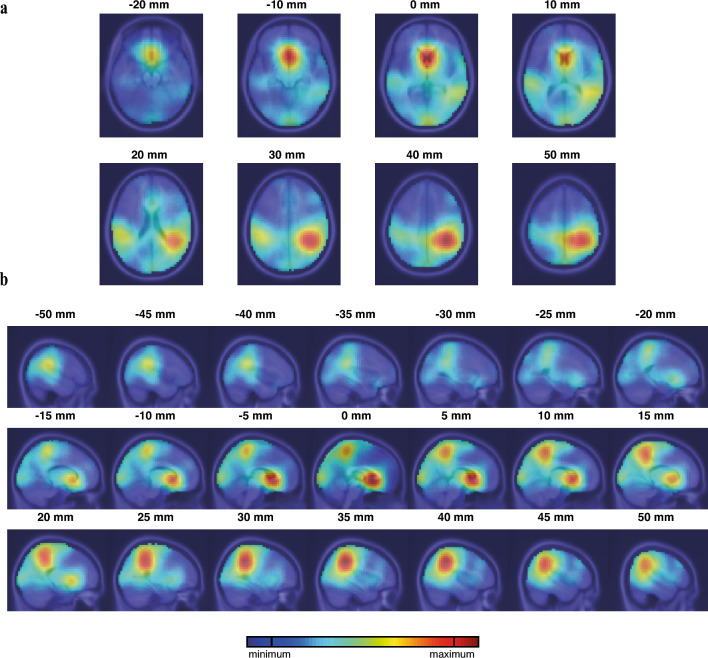
Spatial distribution of the most entrained source densities in the brain. (**a**) Top view slices from the MNI template at z-values ranging from − 20 to 50 mm, showcasing the distribution of source densities. (**b**) Similar to (**a**), but for sagittal slices spanning from left (− 50 mm) to right (+ 50 mm). The color gradient, ranging from red (representing the highest density) to blue (indicating the lowest density), provides a visual representation of the spatial distribution of the most entrained source densities across the entire participant population. The areas exhibiting high densities of entrained sources have overlap with the default mode network.

Using independent component (IC) analysis, we obtain information about the spatial distribution of independent sources within the brain. To account for individual variability in entrainment responses and brain activity, we focus on the most responsive ICs based on their entrainment response (ER) index. The density map in Fig. 3 indicates a notable concentration of sources in the superior parietal lobe, inferior parietal lobe, precuneus, supramarginal, superior frontal, and superior temporal regions which all overlap with active areas within the DMN.

Furthermore, the majority of the identified ICs are located in the parietal regions when considering both the left and right sides together. This observation is consistent with our previous findings, which indicate significant improvements in P2P connectivity compared to no significant improvement in F2F connectivity.

### Connectivity over time: temporal effects of auditory entrainment and group-wise differences

**Figure 4:**
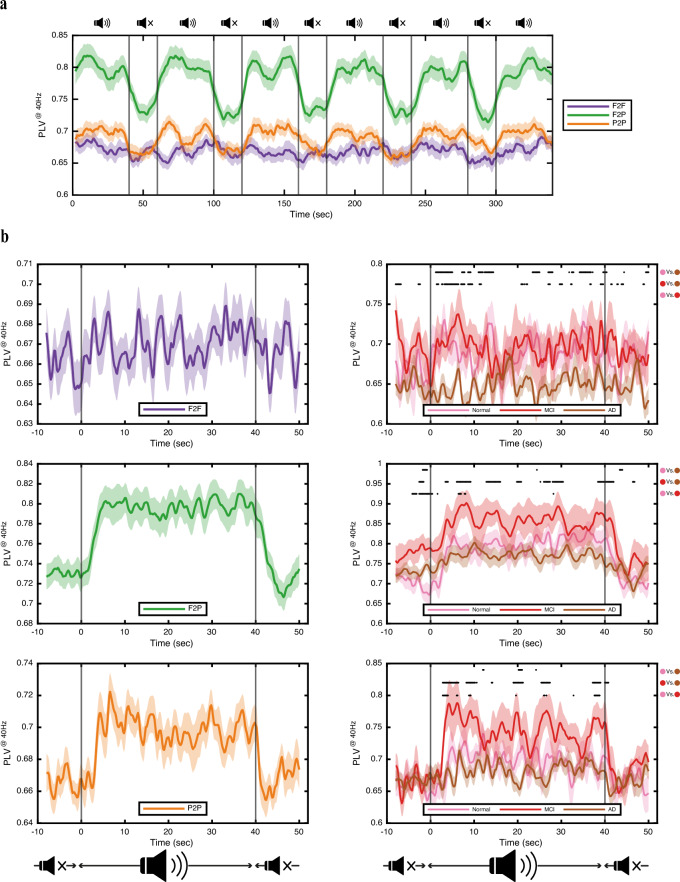
Temporal dynamics of connectivity at 40 Hz and group-wise analysis. (**a**) Connectivity at 40 Hz measured via phase locking value (PLV) over the entire duration of the entrainment task for interregional F2P and intraregional F2F and P2P connections. The PLVs are computed over windows of 2 s in length with 90% overlap. Notice the increasing trend following the onset of auditory stimulation, particularly evident for F2P and P2P connections. However, no cumulative effect is observed over the entire duration of the task. Vertical lines denote the beginning of stimulus and rest cycles, indicated by a speaker icon and a muted speaker icon, respectively. The thick lines represent the mean values across all participants, with shades corresponding to the standard error of the mean. (**b; left**) Similar to (**a**) but depicting the average of all trials locked to the stimulus onset (time zero) and averaged over all participants. Connectivity for each group of sites is plotted separately: F2F in the upper panel, F2P in the middle panel, and P2P in the lower panel. Note the time lag for connectivity to reach its maximum at the onset of stimulus and the subsequent undershoot at the beginning of rest, particularly pronounced for P2P and F2P connections. P2P sites exhibit faster change dynamics compared to F2P sites. (**b; right**) Paired with (**b; left**), but instead of displaying averages for all participants, PLV is averaged over each group of participants separately: Normal, MCI, and AD. Group-wise comparisons are made at each time sample for each pair of groups: Normal vs. AD, MCI vs. AD, and Normal vs. MCI. Statistically significant differences (p-value < 0.05) between each pair of groups are indicated by star signs above the plots. The F2F site demonstrates the most amount of separation between the AD group and the other two groups (MCI and Normal), whereas the F2P and P2P sites display the most amount of separation between the MCI group and the other two groups (AD and Normal). Together, these two observations suggest that intraregional connectivity in the frontal area may serve as an important topographical marker for AD, while cross-regional F2P and intraregional P2P connectivity may serve to indicate the presence of MCI.

Illustrations in Fig. 4 offer a temporal view of the entrainment's impact on local and distant synchronization within the frontoparietal network over the entire duration of the stimulus. To explore temporal fluctuations in PLV during entrainment, we conduct a finer-grained analysis by recalculating PLV at 40 Hz and over 2 s windows with a 90% overlap, revealing dynamic changes displayed in Fig. 4.

Figure 4a unfolds a temporal narrative, showcasing fluctuations in synchronization among different sites throughout the entrainment session. Despite its variability in time, the mean PLV during each stimulus trial consistently exceeds the mean of the rest intervals for both F2P and P2P sites in agreement with observations in Fig. 2. Notably, synchronization between the distant F2P sites consistently shows higher levels throughout the task compared to the intraregional F2F and P2P sites. Furthermore, an increasing trend following the onset of the auditory stimulation is observed particularly in the F2P and P2P connections. One can also note that no cumulative effect is observed across trials and over the entire duration of the task.

Figure 4b illustrates the dynamic evolution of PLV during stimulus trials for the F2F (upper panel), F2P (middle panel), and P2P (lower panel) sites, both across all participants (left panel) and within each group (right panel: Normal, MCI, and AD). Focusing on the F2P and P2P connectivity which demonstrate considerable differences between the stimulus and rest trials, the initiation of each stimulus trial sees a gradual increase in PLV, reaching its maximum value after about 5 s for F2P and about 4 s for P2P sites, indicating a delayed effect for the stimulus to fully impact connectivity enhancement. At stimulus termination, a gradual decrease is observed in PLV, taking about 10 s for F2P and 5 s for P2P sites, during which they return to their initial values after undergoing an undershoot effect. Notably, P2P sites exhibit faster temporal dynamic changes at the onset of stimulus changes compared to F2P sites.

The right panels of Fig. 4b illustrate temporal dynamics of PLV presented separately for each group of participants. All three sites contain temporal instances which display significant differences between pairs of subject groups as indicated on each plot. There are however differences in the performance of the three sites allowing to use them to distinguish between the three subject groups. The F2F sites demonstrate the most amount of separation between the AD group and the other two groups (MCI and Normal). The F2P and P2P sites display the most amount of separation between the MCI group and the other two groups (AD and Normal). Together, these two observations on the performance of PLV in separating the subject groups suggest that intraregional connectivity in the frontal area may serve as an important topographical marker for AD, while cross-regional F2P and intraregional P2P connectivity may serve to indicate the presence of MCI. Notably, while the connectivity performance is markedly lower in AD patients than both the Normal and MCI groups, higher values of connectivity are observed for the MCI groups compared to the Normal group, especially in the F2P and P2P sites. This observation might serve to suggest that higher than normal synchronization in sites involving the parietal area in MCI patients may point to a compensation mechanism to preserve the functionality of the data flow and integration pathways for cognitive operations.

## Discussion

Synchronized oscillations play a crucial role in cognitive functions as they reflect the temporal and spatial coordination of oscillatory activity in different brain regions^51,52^. The disruption of synchronized activity has been observed in dementia and AD, underscoring its significance in cognitive impairments^51,52^. Specifically, the default mode network (DMN), which is involved in self-referential thinking, memory consolidation, and attentional processes, exhibits altered connectivity patterns in individuals with dementia^25,27,53,54^.

The aberrant connectivity within the DMN aligns with the characteristic cognitive deficits observed in dementia, including impairments in memory, attention, and executive functions. The DMN encompasses various brain regions, including the parahippocampal gyrus (PHG), dorsal prefrontal cortex (dPFC), medial prefrontal cortex (mPFC), anterior cingulate cortex (ACC), posterior parietal cortex (PCC), precuneus, and inferior parietal lobe (IPL). The cooperative activity of these regions forms a large-scale network within the brain^53,55,56^.

The localization of sources within the DMN, their entrained state with increased power at 40 Hz during the stimulus, and the observed synchronization among DMN sources collectively support the role of entrainment in triggering brain activity in the regions involved in the DMN and enhancing its functionality. The predominance of posterior ICs further supports the notion that the parietal regions play a crucial role in the entrainment process, potentially influenced by the proximity of the auditory sensory processing cortex in the temporal lobe. These findings have implications for understanding the mechanisms underlying cognitive impairments in dementia and provide insights into potential therapeutic interventions targeting DMN connectivity. Further research is warranted to elucidate the specific mechanisms through which entrainment influences DMN activity and to explore its clinical potential in improving cognitive functions in individuals with dementia and related conditions.

### Exploring the impact of entrainment on the DMN: unraveling the cognitive dynamics of task-negative activity

The interaction between entrainment and the DMN raises the question of why entrainment affects the DMN. The DMN is known for its involvement in task-negative activity, and there appears to be a consistency between the type of stimulation used during the entrainment session and the role of the DMN. During the entrainment session, participants are exposed to sensory stimulation in the form of meaningless sounds, without engaging in specific high-level cognitive tasks. They are simply listening to the sound while their mind can continue its resting state processes. Although the level of attention paid to the stimulus could potentially affect the quality of entrainment and is a topic for future investigation, the sound itself does not contain any meaningful information or require participants to perform task-driven processes.

This scenario can be compared to the experience of listening to the monotonous sound of large fans in a hall. Initially, the sound may be noticeable, but over time, it fades into the background and is no longer consciously perceived. It is only when the fans are switched off that one realizes the presence of the previously unnoticed sound. Such cognitive processes of filtering out and disregarding irrelevant stimuli can be attributed to the DMN. Therefore, the effect of entrainment on the DMN may be justified by considering the DMN’s role in processing and modulating the perception of stimuli that do not require active engagement. However, further research is needed to explore and validate this justification in more depth.

### Entrainment and neural plasticity

New treatment approaches for AD can aim to revive the affected neuronal communication pathways by resuscitating the weakened synapses through entraining the surviving synaptic activity. Entraining the brain in repetitive sessions has been reported to hinder plaque formation^35^. A relevant question is how entrainment is able to enliven the characteristics reflecting the quality of synchronized oscillatory activity.

Neuronal populations engaged in synchronized activity resemble a group of musicians playing in an orchestra producing distinct rhythms, which they tune temporally and collectively by following a reference beat. As synaptic loss extends due to the progress of AD, the production of rhythmic oscillations loses synchronicity across the network as large neuronal populations turn into rather isolated smaller groups which cannot keep track of each other’s rhythms as they did before, leading to arrhythmic brain activity^4^. The entraining stimulant enters the scene as a new conductor (more precisely a metronome), pumping rhythmic beats into the neuronal populations and forcing oscillatory activity at the entrained frequency into synchronicity. Indicating factors for the efficacy of entrainment can therefore be the temporal span and the spatial spread across which synchronized gamma activity is induced by the stimulant. Figure 1b shows the rather large area in which high power activity is observed at the stimulant frequency. Furthermore, the entrained activity maintains a persistent phase during stimulation cycles as evidenced in Fig. 2b, indicating that the induced activity is temporally synchronized. Moreover, the spatial coupling of the induced gamma oscillations in the frontal and parietal areas points to the ability of the entrainment pump to bring DMN as one of the effectual neural circuitry across the brain into synchrony at the target frequency. However, significantly lower levels of synchronization within the frontal region may imply a distinction in spatial synchrony between the entrainability of the frontal and parietal lobes. In addition, the frontoparietal spatial synchrony in the entrained brain mimics the characteristics of long-range communication activities observed in the DMN for the healthy brain which are involved in sensory processing, memory functions, and cognition^53,57–59^.

How can the entrainment of gamma band oscillatory activity lead to the therapeutic effects reported for AD^33–36,39,40,60^? One mechanism which may help explain these effects is the principle of neuroplasticity, which states that the synaptic links between neurons experiencing synchronized activity strengthen over time. Through enticing simultaneous neural activity, entrainment contributes to binding neural pathways and boosting of synaptic weights in neuronal populations that are forced to undergo synchronized activity.

### Entrainability as a predictive indicator of response to treatment in participants

Our study examined the strength of the induced gamma power and individual responsiveness to entrainment, revealing a correlation between entrainment and connectivity in frontoparietal sites. Considering that connectivity between these regions is influenced by dementia progression, we propose the use of the entrainment response (ER) as an index to assess brain synchronization and potentially code the state of the disease.

The ER can serve as a diagnostic tool for evaluating the severity and progression of dementia. By analyzing individual-level data, we can quantify entrainability and determine the degree of synchronization in the brain. This index also offers promise for personalizing therapeutic interventions through optimizing stimulation sessions to maximize the entrainment of brain oscillatory activity from its baseline.

Our findings may suggest that individuals with higher entrainability are more likely to benefit from interventions targeting the DMN, which is associated with cognitive functions. Thus, entrainability can be a valuable predictor of treatment response. Identifying those with higher entrainability may allow for indicating them as potential candidates for interventions aimed at restoring DMN functionality.

### Exploring the effect of auditory entrainment in temporal areas

In our investigation of auditory entrainment, an intriguing observation arises—despite the auditory nature of the stimulus, there appears to be a less pronounced entrainment response in temporal channels, which are  associated with the initial stages of auditory processing. The topographic plots in Fig. 1b illustrate entrainment responses across all unipolar channels, revealing notably higher responses in frontal and parietal lobes compared to other regions. This, however, does not imply a lack of response in the temporal areas but rather a relatively lower level of response there compared to the frontal, parietal, and occipital areas.

In Fig. 3, the distribution of sources (dipoles) associated with cortical activity exhibiting strong entrainment response is shown. The identified dipoles predominantly represent deep sources within the brain, aligned with the DMN. Notably, these sources exhibit an overlap with the temporal area, as depicted in the lateral cuts of the sagittal view or the superior cuts of the top view in Fig. 3. The presence of sources in the temporal areas suggests response activity within this region.

### Limitations and future directions

Our study had some limitations in its scope and implementation. First, since our main objective has been to provide interpretive evidence for the therapeutic effects of gamma entrainment, we limited the scope of our data collection to only one session with multiple trials. This choice was to alleviate the burden of multiple visits to the clinic for our elderly participants. While processing data acquired from one session already provided a multi-faceted interpretation for the mechanisms by which gamma entrainment improves network performance, obviously a longitudinal study is required to draw conclusions on how these network characteristics evolve over time.

Second, as other studies begin to report initial findings from longitudinal assessment of the entrainment-based therapy in humans^47^, important practical questions remain unanswered. For instance, determining the stage of dementia or AD at which gamma entrainment can still be effective in reversing the symptoms of the disease, and characterizing the longevity of the therapeutic improvements after the completion of the therapy, are crucial aspects that require further investigation. Besides addressing these questions, it is essential to evaluate the predictive potential of the ER index in determining the efficacy of the therapy. These questions also pertain to any approach aiming to provide a network-based interpretation of the effects of entrainment, and deserve attention in further related studies.

## Methods

### Participants

Thirty-five volunteers (17 females, 54–89 years of age) were recruited from referrals to the memory clinic of Ziaeian Hospital in Tehran with memory performance complaints. A neuropsychologist from the Department of Geriatric Medicine of Ziaeian Hospital conducted all clinical procedures for this study. The participants’ age, level of education, preferred hand, and smoking history were recorded. Cognitive status was quantified using the mini-mental state examination (MMSE). A neurologist examined the probable AD state in each participant according to the latest guideline of the NIA-AA^61^.

This study was approved by the Review Board of Tehran University of Medical Sciences (Approval ID: IR.TUMS.MEDICINE.REC.1398.524). All methods were performed in accordance with the relevant guidelines and regulations, and all participants provided informed consent before participating and were free to withdraw at any time. To accommodate participants who preferred a shorter duration of data gathering, we designed short and long sessions for entrainment. This approach aimed to minimize inconvenience for the participants who were less inclined to engage in lengthy procedures. Participant demographics, including age and neuropsychological scores, are outlined in Table 1 in Supplementary Material.

General exclusion criteria were: a history of stroke, traumatic brain injury, schizophrenia, major depressive disorders and electroconvulsive therapy (ECT) over the prior 6 months, or other neurodegenerative diseases (Parkinson’s disease, multi-system atrophy, cortico-basal degeneration, progressive supranuclear palsy). Two participants (P6 and P13) were excluded from the study as the diagnosis of their status required further examination not scoped in this study.

### EEG recording and preprocessing

All EEG data were recorded using 19 monopolar channels based on the standard 10/20 system. For the short session, the reference electrodes were placed on the earlobes, while for the long session, referencing was done to the FCz channel. Notably, re-referencing to the average was implemented during preprocessing, ensuring data integrity and minimizing potential interference. The sampling rate was set to 250 Hz, and the impedance of the electrodes was kept under 20 k$\Omega$. During the experiment, participants were seated comfortably with open eyes in a quiet room, and they were instructed to relax their body to avoid muscle artifacts and to move their head as little as possible. Before the main task, a one-minute data was recorded with open eyes for measuring raw resting-state potentials.

Data from all participants were preprocessed identically following Makoto’s preprocessing pipeline^62^: Highpass filtering above 1 Hz; removal of the line noise; rejecting potential bad channels; interpolating rejected channels; re-referencing data to the average; artifact subspace reconstruction (ASR); re-referencing data to the average again; estimating the brain source activity using independent component analysis (ICA); dipole fitting; rejecting bad dipoles (sources) for further cleaning the data. These preprocessing steps were performed using EEGLab^63^ toolbox in MATLAB. Finally, the data (each channel) were normalized to zero mean and unit variance for further analysis and making comparison among participants.

### Source (dipole) localization

To localize dipoles within the brain tissue, we utilized the DIPFIT plugin in EEGLAB. This involved using a boundary element model (BEM) template for the head, which consisted of three 3D surfaces representing the skin, skull, and cortex. These surfaces were derived from the Montreal Neurological Institute (MNI) canonical template brain, providing a high-quality anatomical MRI of a representative individual.

To ensure accurate registration of the electrode positions on the template, we performed co-registration procedures to align the electrode positions with the selected head model. Subsequently, we localized the equivalent dipole source for each independent component (IC) obtained during the preprocessing stage. In cases where IC scalp maps appeared bilaterally symmetric, we employed a bilateral dipole fitting approach^64^.

Furthermore, we calculated the entrainment response (ER) for each IC, which allowed us to quantitatively assess the power of entrainment. We associated each dipole source involved in the brain entrainment process with the ER value for the related IC.

### Bipolar setup: assessing interregional and intraregional sites

The EEG data were initially recorded in a unipolar fashion from 19 positions, with all channels sharing the same reference even after re-referencing the data to the average. This recording setup allowed us to capture the oscillatory activity of each underlying lobe relative to a common reference point. However, bipolar EEG data involves the calculation of relative electrical activity between two channels, resulting in channels that do not share a common reference. This approach enables a more specific analysis of the relative activity between two separate channels and facilitates the interpretation of data pertaining to the specific regions from which they were recorded.

For our synchronization analysis, we employed a bipolar setup to investigate the interaction of recorded activity between frontal and parietal regions. The bipolar sites were calculated in a manner that allowed us to examine both intraregional bipolar activity within the frontal and parietal regions as well as interregional activity between the frontal and parietal areas.

The intraregional bipolar activity within the frontal region (F2F) was computed by subtracting the recorded activity of one channel from another within the set of frontal channels. Specifically, Fp1, Fp2, F3, Fz, and F4 were selected as the monopolar channels, and their pairwise subtractions resulted in 10 bipolar F2F sites. Similarly, the intraregional bipolar activity within the parietal region (P2P) was obtained by subtracting the activity of two selected monopolar channels within the set of parietal channels. For this, pairwise subtractions between channels P3, Pz, P4, O1, and O2 yielded 10 bipolar P2P sites. In addition, to assess the interregional activity between the frontal and parietal regions (F2P), we subtracted the EEG data recorded from a frontal channel and a parietal channel. This calculation resulted in 25 different F2P sites. Table 2 in Supplementary Material provides a summary of all the aforementioned bipolar sites, specifying the combinations of channels used for each site. Additionally, Fig. 2a visually represents the unipolar channels associated with each site, thereby illustrating the specific locations of the bipolar sites. These visual representations were generated using BrainNet Viewer^65^.

This bipolar setup allows us to analyze the specific interactions and relative activity between different brain regions, providing insight into the synchronization patterns of short-range and long-range connections.

### Entrainment session and auditory stimulation

Each session involved the presentation of a multi-trial auditory stimulus while simultaneously recording EEG data from the participant. To deliver the auditory stimulus, two speakers were placed in front of the participant, 50 cm apart from each other and directly pointed at the participant’s ears at a distance of 50 cm. The sound intensity was around – 40 dB within a fixed range for all participants. To ascertain adequate hearing ability of the participants and to ensure individual comfort, each participant was asked before commencing the task if the sound was at a comfortable level, and adjustments were made to the volume. The auditory stimulus was a 5 kHz carrier tone amplitude modulated with a 40 Hz rectangular wave (40 Hz On and Off cycles). Since a 40 Hz tone cannot be easily heard, the 5 kHz carrier frequency was used to render the 40 Hz pulse train audible. In order to minimize the effect of the carrier sound, the duty cycle of the modulating 40 Hz waveform was set to 4% (1 ms of the 25 ms cycle was On). The auditory stimulant was generated in MATLAB and played as a .wav file. This file consisted of multiple trials, with each trial lasting 40 s and interleaved by 20 s of rest (silence). The short session included six trials, while the long session comprised ten trials of the stimulus.

### Entrainment score/response (ES/ER)

To quantitatively analyze the entrainment of each channel/IC throughout the task, we introduced the entrainment score (ES) as a parameter that measures the degree of entrainment at the 40 Hz stimulating frequency.

During the stimulus presentation trials, we observe sharp and distinct peaks at 40 Hz in EEG signals. The power of different frequency bins can vary among subjects, even after normalizing data for inter-participant comparability. This implies that a high power at 40 Hz in stimulus trials does not necessarily indicate a pronounced peak at the entraining frequency. To quantify the presence and sharpness of the 40 Hz peak, we used z-scoring in the calculation of the ES. This involves applying z-score calculations to frequency samples of the power spectral density (PSD) within the range of 38–42 Hz rather than applying it over trials or participants. The objective is to understand how many standard deviations the power of the 40 Hz peak exceeds the average power of the frequency bins in the neighboring range. The z-score value at 40 Hz serves as the ES and hence is calculated independently for each participant. For paired analysis between the stimulus and rest conditions, the ES was calculated for each trial, and a statistical test was applied.

To assess the impact of stimulus-driven entrainment, we defined the entrainment response (ER) index as the difference between the ES values during the stimulus and rest intervals. This enables us to specifically investigate the changes in entrainment response induced by stimulation. It is important to note that almost the entire cortex undergoes entrainment (Fig. 1b), but for ranking purposes, we focus on the entrainment response calculated for channel Fz, which was recognized as one of the most responsive channels to entrainment among the entire study population.

### Phase locking value (PLV)

After applying a narrow bandpass filter centered around the frequency of interest, we utilized the Hilbert transform to extract phase information, a method well-defined for narrowband signals but problematic for wideband signals. Given our focus on investigating the response at the 40 Hz frequency, we used a narrowband filter with a bandwidth of 0.5 Hz (on both sides) of the type of a second-order Butterworth filter. This choice minimizes phase distortion due to its maximally flat magnitude response and near-linear phase response. Then, the recorded data from each bipolar site were segmented into 20 s windows (or windows of 2 s for generating plots in Fig. 4). To evaluate phase synchronization between two distinct sites (S1 and S2 as an example) within each window, we averaged the S1–S2 phase-locking vectors of all temporal samples in that window. Denoting $\phi_{S1} \left( t \right)$ and $\phi_{S2} \left( t \right)$ as the phases of the target frequency bin oscillations at the S1 and S2 sites at time $t$, respectively, the PLV of the two sites was computed as:

1 $$PLV = \frac{1}{N}\left| {\mathop \sum \limits_{t = 1}^{N} e^{{j\left( {\phi_{S1} \left( t \right) - \phi_{S2} \left( t \right)} \right)}} } \right|,$$

where $N$ is the total number of temporal samples. The procedure was replicated across a spectrum of frequencies, ranging from 1 to 45 Hz (with increments of 0.5 Hz), serving as the central frequency for a second-order Butterworth filter. This approach allows us to obtain PLVs for different frequency bins.

The PLV calculation for both the stimulus and rest cycles involved averaging the PLVs obtained from related trials. However, it is crucial to acknowledge the "common reference" problem associated with PLV^66^. The PLV calculates the correlation of two signals with zero-lag, making it susceptible to spurious results when applied to channels with the same reference^66^. The common reference problem arises from the activity of the reference electrode, which is present in the recorded signals of the two channels and can inherently contribute to indices that measure the synchronization of their activity.

To mitigate this issue, it is recommended to calculate PLV between bipolar sites that do not share any common channels^66^. This is the reason we focused on investigating connectivity between different sites rather than different unipolar channels. To assess the synchronized activity of interregional and intraregional sites, the PLV was separately calculated for each pair within the three interaction sets: F2F, F2P, and P2P. The reported PLV for each set represents the average PLV calculated for the paired sites belonging to that set. In the averaging procedure, we took the common reference problem into consideration and avoided including paired sites with a common channel.

### Statistical analyses

We employed paired-sample t-tests to assess the statistical significance of the differences between stimulus and rest conditions across the entire population. This choice was made because the number of participants in the study exceeds 30, allowing us to reasonably assume a normal distribution. However, for group-wise analyses, a non-parametric approach, specifically the Wilcoxon rank-sum test, was preferred, due to the limited number of samples per group.

The bar plots present the mean values along with the standard error of the mean (SEM) indicated by black lines. The individual samples are represented by empty circles. The paired relationship of each sample is depicted by gray lines. A p-value of less than 0.05 was considered statistically significant. The significance levels are denoted as follows: *, p < 0.05; **, p < 0.01; ***, p < 0.001. Additionally, the correlation values reported are Pearson correlations, which indicate the strength and direction of the relationships between variables.

### Supplementary Information

Supplementary Information.

## Data Availability

The dataset generated and analyzed in the current study is available at the OpenNeuro repository accessible at the following link: 10.18112/openneuro.ds005048.v1.0.0.
