# Supplementary material for: Auditory gamma-band entrainment enhances default mode network connectivity in dementia patients
Source: Sci Rep. 2024 Jun 7;14:13153. doi: 10.1038/s41598-024-63727-z (PMC11161471; doi:10.1038/s41598-024-63727-z)
Supplement: Supplementary file 1 — Supplementary Information. [file 41598_2024_63727_MOESM1_ESM.pdf]

Supplementary Material

Auditory gamma-band entrainment enhances default mode network connectivity in dementia patients

Mojtaba Lahijanian[1], Hamid Aghajan[1*], Zahra Vahabi[2]
1 Department of Electrical Engineering, Sharif University of Technology, Tehran, Iran
2 Department of Geriatric Medicine, Ziaeian Hospital, Tehran University of Medical Sciences, Tehran, Iran
***** Corresponding Author; Email: aghajan@ee.sharif.edu

Table 1. General information of participants.

Index	Participant code	MMSE score	Gender	Age	Dementia state	Inclusion
1	P1	28	Male	65	Normal	Yes
2	P2	23	Female	70	Mild AD	Yes
3	P3	27	Male	75	Normal	Yes
4	P4	21	Male	82	Mild AD	Yes
5	P5	21	Male	75	Mild AD	Yes
6	P6	-	Female	69	-	No
7	P7	19	Male	89	Mild AD	Yes
8	P8	24	Male	70	Normal	Yes
9	P9	26	Female	57	Normal	Yes
10	P10	28	Male	81	Normal	Yes
11	P11	20	Female	88	Mild AD	Yes
12	P12	30	Female	63	Normal	Yes
13	P13	-	Male	60	-	No
14	P14	18	Female	79	Mild AD	Yes
15	P15	19	Female	77	Mild AD	Yes
16	P16	25	Male	68	Mild AD	Yes
17	P17	25	Male	65	MCI	Yes
18	P18	18	Female	76	MCI	Yes
19	P19	13	Female	82	MCI	Yes
20	P20	13	Female	82	Mild AD	Yes
21	P21	30	Male	78	Mild AD	Yes
22	P22	29	Female	67	Normal	Yes
23	P23	14	Female	75	Mild AD	Yes
24	P24	23	Male	81	Moderate AD	Yes
25	P25	24	Female	68	Normal	Yes
26	P26	28	Male	54	Normal	Yes
27	P27	23	Male	64	MCI	Yes
28	P28	26	Male	60	MCI	Yes
29	P29	16	Male	85	Mild AD	Yes
30	P30	22	Male	71	Mild AD	Yes
31	P31	26	Female	59	Normal	Yes
32	P32	21	Female	71	MCI	Yes
33	P33	15	Female	83	Mild AD	Yes
34	P34	20	Female	78	Mild AD	Yes
35	P35	21	Male	77	Mild AD	Yes

Table 2. Frontal and parietal intraregional and frontoparietal interregional bipolar sites and their associated unipolar channels.

Index	Site	1st channel	2nd channel	Group
1	S1	Fp1	Fp2	F2F
2	S2	Fp1	F3	F2F
3	S3	Fp1	Fz	F2F
4	S4	Fp1	F4	F2F
5	S5	Fp1	P3	F2P
6	S6	Fp1	Pz	F2P
7	S7	Fp1	P4	F2P
8	S8	Fp1	O1	F2P
9	S9	Fp1	O2	F2P
10	S10	Fp2	F3	F2F
11	S11	Fp2	Fz	F2F
12	S12	Fp2	F4	F2F
13	S13	Fp2	P3	F2P
14	S14	Fp2	Pz	F2P
15	S15	Fp2	P4	F2P
16	S16	Fp2	O1	F2P
17	S17	Fp2	O2	F2P
18	S18	F3	Fz	F2F
19	S19	F3	F4	F2F
20	S20	F3	P3	F2P
21	S21	F3	Pz	F2P
22	S22	F3	P4	F2P
23	S23	F3	O1	F2P
24	S24	F3	O2	F2P
25	S25	Fz	F4	F2F
26	S26	Fz	P3	F2P
27	S27	Fz	Pz	F2P
28	S28	Fz	P4	F2P
29	S29	Fz	O1	F2P
30	S30	Fz	O2	F2P
31	S31	F4	P3	F2P
32	S32	F4	Pz	F2P
33	S33	F4	P4	F2P
34	S34	F4	O1	F2P
35	S35	F4	O2	F2P
36	S36	P3	Pz	P2P
37	S37	P3	P4	P2P
38	S38	P3	O1	P2P
39	S39	P3	O2	P2P
40	S40	Pz	P4	P2P
41	S41	Pz	O1	P2P
42	S42	Pz	O2	P2P
43	S43	P4	O1	P2P
44	S44	P4	O2	P2P
45	S45	O1	O2	P2P
